# MuSK-Associated Myasthenia Gravis: Clinical Features and Management

**DOI:** 10.3389/fneur.2020.00660

**Published:** 2020-07-23

**Authors:** Carmelo Rodolico, Carmen Bonanno, Antonio Toscano, Giuseppe Vita

**Affiliations:** Department of Clinical and Experimental Medicine, University of Messina, Messina, Italy

**Keywords:** muscle-specific tyrosine kinase, atypical onset, tongue atrophy, MuSK-MG therapy, rituximab

## Abstract

Muscle-specific tyrosine kinase (MuSK) myasthenia gravis (MG) is a rare, frequently more severe, subtype of MG with different pathogenesis, and peculiar clinical features. The prevalence varies among countries and ethnic groups, affecting 5–8% of all MG patients. MuSK-MG usually has an acute onset affecting mainly the facial-bulbar muscles. The symptoms usually progress rapidly, within a few weeks. Early respiratory crises are frequent. The disease may lead to generalized muscle weakness up to muscle atrophy. The main bulbar involvement, the absence of significant thymus alterations, and the association with HLA class II DR14, DR16, and DQ5 alleles have been confirmed. Atypical onset, such as ocular involvement, lack of symptom fluctuations, acetylcholinesterase inhibitors failure, and negative results of electrophysiologic testing, if not specifically performed in the mainly involved muscle groups, makes MuSK-MG diagnosis challenging. In most cases, steroids are effective. Conventional immunosuppressants are not commonly able to replace steroids in maintaining a satisfactory long-term control of symptoms. However, the majority of MuSK-MG patients are refractory to treatment. In these cases, the use of rituximab showed promising results, resulting in sustained symptom control.

## Introduction

In 2001, serum antibodies against muscle-specific tyrosine kinase (MuSK-Abs) were identified for the first time as cause of myasthenia gravis (MG) (1), opening the way to the description of a distinct peculiar subtype of MG disease (2–5). A reliable neuromuscular junction (NMJ) transmission is guaranteed by both morphological NMJ appropriate structure and NMJ transmission efficacy. NMJ transmission efficacy is strictly related to the “safety factor,” which refers to the ability of the NMJ to remain effective under several conditions. This is possible mainly because each nerve impulse releases more transmitter than is required to excite the muscle fiber, ensuring that the transmission does not fail (6). The role of muscle-specific tyrosine kinase (MuSK) in determining NMJ efficacy has been recently clarified (7). A tetrameric complex on the postsynaptic membrane results from the association between MuSK and the low-density lipoprotein receptor-related protein 4 (LRP4). The MuSK–LRP4 tetramer is phosphorylated by agrin and recruits downstream of kinases 7, which further enhances MuSK activation for postsynaptic differentiation and acetylcholine receptor (AChR) clustering. Furthermore, an interaction between MuSK and matrix proteins, such as collagen Q (ColQ), which contributes to synapsis stabilization, has been demonstrated *in vitro* (8, 9). Recently, Huijbers et al. confirmed MuSK-Abs as pathogenetic (10). MuSK-Abs belongs mostly to the IgG4 class of immunoglobulins, which acts by the direct inhibition of protein function. In particular, MuSK-Abs interfere with MuSK–LRP4 complex and, consequently, AChR clustering is inhibited (11). The aim of this mini-review is to report on the epidemiological and major clinical features, diagnostic approach, and treatment of MuSK-MG subtype.

## Epidemiology

MuSK-MG is reported in about 5–8% of MG patients. Its prevalence varies among countries and ethnic groups, with a higher percentage in Southern Europe, and it is clearly predominant in females, actually constituting more than 70% of patients in all studies reviewed (9, 12).

The disease has an early age of onset, with a peak of incidence in the late 3rd decade, and it rarely occurs after 70 years of age. Cohorts from different countries confirm the association with HLA class II DR14, DR16, and DQ5 (9). No significant thymus alterations have been reported in MuSK-MG patients as related to the disease (9, 12, 13).

## Clinical Features

A peculiar clinical onset picture has been described from several groups for MuSK-MG. The disease typically has an acute onset, with rapid progression within a few weeks. In the majority of cases, bulbar involvement appears in the first stage and the presenting symptoms are ptosis and diplopia.

However, some peculiarities have been demonstrated about ocular manifestations which are observed in the early stages of the disease, consisting in symmetrical ophtalmoparesis of horizontal gaze and, more rarely, of vertical gaze with rapid remittance of diplopia. Furthermore, the typical fluctuation of myasthenic symptoms may not be evident in MuSK-MG patients. Commonly, a purely ocular onset generalizes in 2–3 weeks (14–17).

Bulbar impairment has been demonstrated in up to 80% of MuSK-MG patients, consisting of dysarthria, dysphonia with nasal voice, dysphagia, and masticatory difficulty. Bulbar onset is usually related to rapid deterioration, frequently leading to respiratory crisis. Generalized weakness and fatigue have also been described as onset syndrome, resembling anti-AChR-associated MG (AChR-MG). Furthermore, MuSK-MG patients have a higher risk of myasthenic crisis (3). Usually, axial muscle weakness involves neck extensor, which may present as head drop, and it can be the only presenting sign, without bulbar involvement. Neck extensor weakness is more frequent in MuSK- MG, whereas neck flexors could be only mildly involved (18).

**Figure 1:**
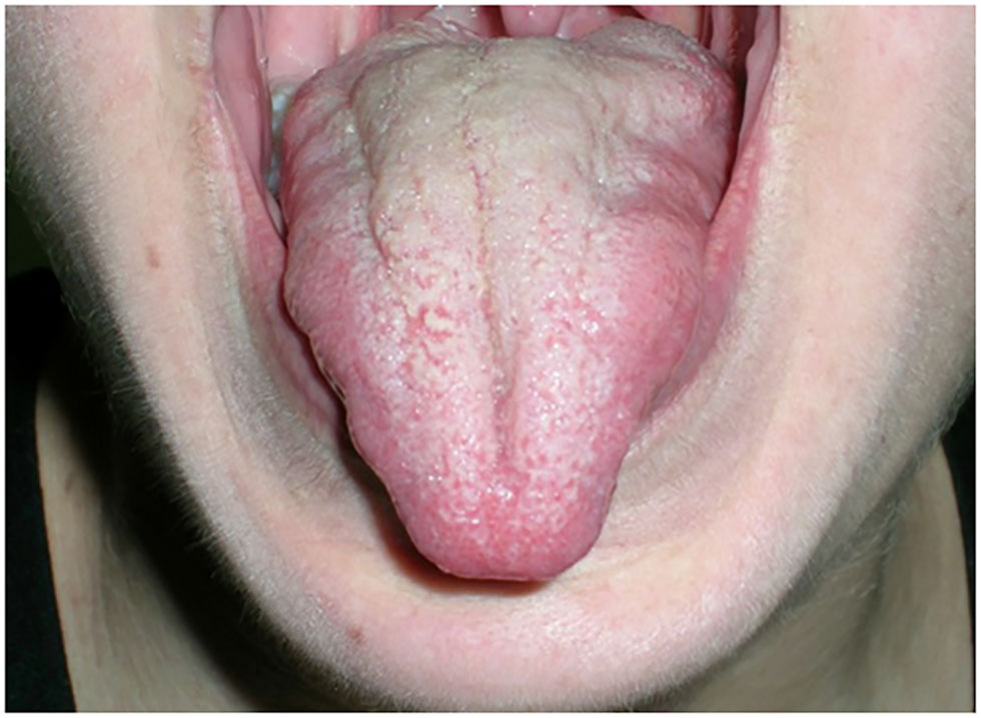
Tongue atrophy in a young woman with MuSK-MG.

An unusual but distinct feature of MuSK-MG is muscle atrophy. In particular, the mainly involved muscle groups are facial muscles and the tongue (Figure 1). Muscular atrophy can also be observed at shoulder girdle muscles, limb, and paraspinal muscles, resulting in severe scoliosis, as reported in a few cases in literature (19).

Electromyography (EMG) on atrophic muscles reveals a myopathic pattern and magnetic resonance imaging confirms muscle thinning and documents fatty replacement. There are evidences that corticosteroid treatment can improve muscle wasting; however, in some cases, atrophy becomes chronic and a significant cause of severe disability (20). The majority of MuSK-MG patients do not present relevant thymus alterations (21, 22). Hyperplasia is rarely described. Case reports incidentally documented thymoma treated with thymectomy (23). There are few data and no consensus on the role of thymectomy in MuSK-MG. In AChR-MG, a randomized, controlled trial of thymectomy in non-thymomatous acetylcholine receptor patients demonstrated a significant improvement in clinical outcomes after thymectomy, as well as a decreased requirement for immunosuppression (24). Conversely, available studies on thymectomy in MuSK-MG outline a limited improvement in clinical outcomes or immunosuppression management after thymectomy (21–24). Moreover, it has been reported that the outcome in MuSK-MG after thymectomy may not be beneficial (25). Therefore, thymectomy in MuSK-MG should not be considered as a therapeutic option.

## Diagnostic Approach

MuSK-MG diagnosis might be challenging. In fact, muscle atrophy, dysphagia, dysarthria, and neck extensor weakness as onset clinical picture may be easily misdiagnosed, for example, with bulbar onset of amyotrophic lateral sclerosis, oculopharyngeal muscular dystrophy, and mitochondrial myopathy. The diagnostic procedure includes MuSK-Ab testing, edrophonium/neostigmine test, and electroneurophysiological studies such as repetitive nerve stimulation (RNS), single-fiber electromyography (SFEMG), and needle EMG.

A positive result for MuSK-Ab, sustained by clinical evidences, supports the diagnosis of MuSK-MG. Detection of MuSK-Ab is usually a second step for AChR-Abs-negative patients or individuals positive for AChR-Ab who do not respond to treatment. It has been recently proposed that radio immunological assay-negative MG sera should be tested for IgG-specific antibodies by MuSK-cell-based assay to increase the detection of antibodies (26). Edrophonium or neostigmine tests, although non-routine, resulted positive in 40–75% of MuSK-MG patients; however, these tests demonstrated a higher sensitivity (97–100%) for AChR-MG diagnosis (27).

RNS sensitivity appears to be lower in MuSK-MG compared with AChR-MG, especially when performed on distal limb muscles. However, it has been reported that it is possible to increase the diagnostic sensitivity of RNS in MuSK-MG by testing proximal muscles, in particular the facial muscles, reaching a diagnostic sensitivity of 75–85% (28, 29). In AChR-MG, RNS usually show a partial recovery of the compound muscle action potential amplitude after a transient decrement during the first responses to low-frequency RNS (U-shaped pattern), not reported in MuSK-MG. On the contrary, a progressive decremental pattern after the fourth or fifth stimulation is typically revealed in Lambert–Eaton myasthenic syndrome (LEMS). It has been demonstrated that a similar pattern is usually found also in MuSK-MG, probably due to an underlying presynaptic dysfunction in MuSK-MG patients, as in the LEMS ones (28).

Needle EMG in patients with MuSK-MG may show myopathic features, rarely observed in AChR-MG, in particular in the facial muscles. These non-specific findings only partially contribute to define diagnosis (26). In cases with evocative clinical manifestations of MuSK-MG, associated with borderline antibody values, SFEMG is mandatory to diagnose MuSK-MG. Furthermore, it is worth to underline the importance to focus SFEMG on the mainly affected muscles to precociously detect alterations. In fact, in MuSK-MG, SFEMG of cervical paraspinals, deltoid, frontalis, and orbicularis oculi, which are usually the first and more frequently involved muscles, may be noticeably abnormal since the beginning of the disease. These patients may conversely have normal jitter in clinically uncompromised muscles (30). Stickler et al. reported cases of normal jitter in the extensor digitorum muscle and frontalis but markedly increased jitter with blocking in neck extensors (29). Cases with normal SFEMG at orbicularis oculi but with abnormal jitter in paraspinal muscles have been described by Padua et al. (31).

## Treatment

Long-term pharmacological treatment is usually required to achieve an effective control of symptoms in MuSK-MG; however, it could be at least challenging.

The symptomatic treatment with acetylcholinesterase inhibitors is generally unsatisfactory and may be deleterious in MuSK-MG. Moreover, the response to pyridostigmine standard doses, used for AChR-MG, lacks efficacy and has poor tolerance because of side effects (26). Among symptomatic drugs for MuSK-MG, recently 3,4-diaminopyridine (3,4-DAP), ephedrine, and albuterol have been considered. The use of 3,4-DAP in MuSK- MG patients has been described as mildly to moderately effective, with no remarkable side effects (32). There is only a report demonstrating a clinical improvement in MuSK-MG due to the administration of both ephedrine and albuterol, two sympathomimetics agents commonly used to treat some phenotypes of congenital myasthenic syndromes (33).

### Immunosuppression

Immunosuppression still represents the mainstay of therapy for MuSK-MG. It is well-known that steroids have a prompt and effective response, but they are burdened by long-term side effects.

A high dose of prednisone, in combination with plasma exchange, is generally recommended for patients experiencing life-threatening weakness or suffering from severe disease deterioration. In these patients, intravenous immunoglobulin should also be considered (27).

Traditional immunosuppressants (azathioprine, mycophenolate, tacrolimus, methotrexate, and cyclosporine), in common clinical practice, have been administered with success in MuSK-MG patients as steroid-sparing agents, but it is usually more difficult to achieve and to ensure long-term and complete control of symptoms (34). It is important to consider that 10–15% of MuSK- MG patients have a refractory disease or suffer from relapses on tapering immunosuppressive medication.

The management of this percentage of patients who do not respond to steroids or traditional immunosuppressants is often difficult. In the previous years, clinical trials and evidences from observational prospective studies encourage the use of monoclonal Ab such as rituximab (RTX), a chimeric anti-CD20 monoclonal Ab (35–37). A significant number of MuSK-MG patients showed a greater and sustained improvement of symptoms after RTX administration, compared to those patients who do not receive RTX administration (37). Immunosuppressants can be reduced or even stopped (37). Topakian et al. confirmed the safety and efficacy of RTX in a large cohort of both AChR-MG and MuSK-MG patients; furthermore, these authors demonstrated a significantly higher rate of remission in patients with MuSK-MG compared to AChR-MG ones (38).

In light of common clinical practice and of the above-mentioned results, a recent consensus recommends RTX as an early therapeutic option in MuSK-MG, suggesting its possible role as a steroid-sparing agent since the beginning of the disease (39). RTX has a good safety profile; however, side effects such as myocardial infarction, spondylodiscitis, agranulocytosis, and two cases of progressive multifocal leukoencephalopathy in MG patients have been reported (40, 41).

## Summary

MuSK-MG is a distinctive, frequently more severe, subtype of MG. Onset is usually acute and typically bulbar, with rapid progression of symptoms within a few weeks. Clinical presentation can be atypical: neck weakness, for example, as onset symptom could be misleading, causing a delay in diagnosis. MuSK-Ab testing confirms the diagnosis when the clinical picture is highly suggestive. SFEMG plays an important role in diagnosing MuSK-MG, and we underline the importance to focus it on the mainly affected muscles to precociously detect alterations.

Response to treatment is often different from that expected in MG patients and achieving a regression of symptoms could be quite challenging. Among immunotherapies, prednisone, plasmapheresis, and RTX are the cornerstones of treatment for MuSK-MG. The main features of MuSK-MG are summarized and compared to the main other subtypes of MG (AChR-MG and LRP4-MG) in Table 1.

**Table 1:** Clinical features and management of MG subtypes.

	**AChR-MG**	**MuSK-MG**	**LRP4-MG**
**CLINICAL FEATURES** **(****9****–****13****)**			
Age of onset	Early onset <50 years	3rd decade	Any
	Late onset ≥50 years		
Sex prevalance	Early onset: female	Female	Female
	Late onset: male		
HLA associations	DRB1*01 DRB1*03, B*08, DRB1*09, DR2, and B7A1	DRB1*14, DRB1*16, and DQB1*05	-
Clinical features	Variable	Bulbar impairment, neck extensor weakness, muscle atrophy Higher frequency of myasthenic crisis	Variable
Thymus	Hyperplasia, AB, and B thymoma	Normal	Rare hyperplastic changes
**ELECTROPHYSIOLOGICAL PROFILE** **(****42****–****44****)**			
SFEMG	Frequently positive (~90%) even in non-affected muscles	~80% positive in affected muscles	Rarely positive
**RESPONSE TO TREATMENT** **(****14**, **26****)**			
AChE-Is	Effective	No benefit, several side effects	Effective
Short-term immunotherapy	Effective PE and IVIG	Effective PE Effective IVIG (possibility of non-responders, IVIG > PE)	Effective PE and IVIG
Long-term immunotherapy	Good control achieved with PDN, AZA (or other traditional immunosuppressant)	Partial answer, difficulty to achieve symptoms control with PDN/AZA Rituximab as effective emerging drug for long-term immunotherapy	Good control achieved with PDN, AZA (or other traditional immunosuppressant)

*AChR-MG, anti-acetylcholine receptor Myasthenia Gravis, MuSK-MG, anti-Muscle specific tyrosine kinase Myasthenia Gravis; LRP4-MG, anti- low-density lipoprotein receptor– related protein 4 Myasthenia Gravis; SFEMG, Single-Fiber electromyography; AChE-Is, acetylcholinesterase inhibitors; PE, plasma exchange; IVIG, Intravenous immunoglobulin; PDN, prednisone; AZA, Azathioprine*.

## Consent for Publication

Written informed consent was obtained from the patient to acquire and publish photos.

## Author Contributions

CR conceived the review. CR and CB were equally involved in literature search, figure and table preparation, and drafted and wrote the manuscript. AT and GV reviewed and revised the final draft of the manuscript. All authors have both approved the submitted version of the manuscript and agreed to be personally accountable for their own contributions.

## Conflict of Interest

The authors declare that the research was conducted in the absence of any commercial or financial relationships that could be construed as a potential conflict of interest.
